# Text Messaging as a Screening Tool for Depression and Related Conditions in Underserved, Predominantly Minority Safety Net Primary Care Patients: Validity Study

**DOI:** 10.2196/17282

**Published:** 2020-03-26

**Authors:** Haomiao Jin, Shinyi Wu

**Affiliations:** 1 Suzanne Dworak-Peck School of Social Work University of Southern California Los Angeles, CA United States; 2 Edward R Roybal Institute on Aging University of Southern California Los Angeles, CA United States; 3 Daniel J Epstein Department of Industrial and Systems Engineering University of Southern California Los Angeles, CA United States

**Keywords:** depression, diabetes mellitus, comorbidity, screening, primary care, health information technology, mobile health, text messaging, patient reported outcome measures

## Abstract

**Background:**

SMS text messaging is an inexpensive, private, and scalable technology-mediated assessment mode that can alleviate many barriers faced by the safety net population to receive depression screening. Some existing studies suggest that technology-mediated assessment encourages self-disclosure of sensitive health information such as depressive symptoms while other studies show the opposite effect.

**Objective:**

This study aimed to evaluate the validity of using SMS text messaging to screen depression and related conditions, including anxiety and functional disability, in a low-income, culturally diverse safety net primary care population.

**Methods:**

This study used a randomized design with 4 study groups that permuted the order of SMS text messaging and the gold standard interview (INTW) assessment. The participants for this study were recruited from the participants of the prior Diabetes-Depression Care-management Adoption Trial (DCAT). Depression was screened by using the 2-item and 8-item Patient Health Questionnaire (PHQ-2 and PHQ-8, respectively). Anxiety was screened by using the 2-item Generalized Anxiety Disorder scale (GAD-2), and functional disability was assessed by using the Sheehan Disability Scale (SDS). Participants chose to take up the assessment in English or Spanish. Internal consistency and test-retest reliability were evaluated by using Cronbach alpha and intraclass correlation coefficient (ICC), respectively. Concordance was evaluated by using an ICC, a kappa statistic, an area under the receiver operating characteristic curve (AUROC), sensitivity, and specificity. A regression analysis was conducted to examine the association between the participant characteristics and the differences in the scores between the SMS text messaging and INTW assessment modes.

**Results:**

Overall, 206 participants (average age 57.1 [SD 9.18] years; females: 119/206, 57.8%) were enrolled. All measurements except the SMS text messaging–assessed PHQ-2 showed Cronbach alpha values ≥.70, indicating acceptable to good internal consistency. All measurements except the INTW-assessed SDS had ICC values ≥0.75, indicating good to excellent test-retest reliability. For concordance, the PHQ-8 had an ICC of 0.73 and AUROC of 0.93, indicating good concordance. The kappa statistic, sensitivity, and specificity for major depression (PHQ-8 ≥8) were 0.43, 0.60, and 0.86, respectively. The concordance of the shorter PHQ-2, GAD-2, and SDS scales was poor to fair. The regression analysis revealed that a higher level of personal depression stigma was associated with reporting higher SMS text messaging–assessed PHQ-8 and GAD-2 scores than the INTW-assessed scores. The analysis also determined that the differences in the scores were associated with marital status and personality traits.

**Conclusions:**

Depression screening conducted using the longer PHQ-8 scale via SMS text messaging demonstrated good internal consistency, test-retest reliability, and concordance with the gold standard INTW assessment mode. However, care must be taken when deploying shorter scales via SMS text messaging. Further regression analysis supported that a technology-mediated assessment, such as SMS text messaging, may create a private space with less pressure from the personal depression stigma and therefore encourage self-disclosure of depressive symptoms.

**Trial Registration:**

ClinicalTrials.gov NCT01781013; https://clinicaltrials.gov/ct2/show/NCT01781013

**International Registered Report Identifier (IRRID):**

RR2-10.2196/12392

## Introduction

Depression is an underdiagnosed comorbidity that can negatively affect functional status, morbidity/mortality, and cost for the treatment of chronic illnesses, such as diabetes [1-5]. Depression screening is an effective approach to reduce the rate of undiagnosed depression and provide timely treatment for patients [6]. On the basis of the growing evidence for the benefits of depression screening, the US Preventive Services Task Force recommends depression screening for every adult in the 2016 update of the clinical guidelines [6].

Nevertheless, there are significant barriers for adopting mass depression screening, particularly in underserved, predominantly minority patients with chronic illnesses. This patient population has an increased risk of depression and often prefers safety net primary care over specialty psychiatric care when seeking mental health care [7-9]. However, safety net primary care providers often find themselves lacking time and resources to address mental health issues on top of managing other medical conditions such as diabetes [10-13]. In addition, minority patients are less likely to voluntarily report depressive symptoms. They may view depression as a moral weakness or character flaw rather than an illness and may be more likely to ascribe symptoms of depression to a physical illness [14]. Therefore, underserved minority patients in safety net care systems often miss out on screening and are less than half as likely as non-Hispanic whites to receive any depression care or guideline-level depression care [11,15]

The increasing usage of mobile services, particularly SMS text messaging, provides opportunities to overcome the barriers for adopting universal depression screening in underserved populations. The use of SMS text messaging is highly prevalent globally; among the 4 billion mobile phones in use, 3.05 billion (75%) are SMS text messaging–enabled [16]. In the United States, texting among adult mobile users is higher among minorities such as Hispanics/Latinos (83%) than non-Hispanic whites (70%) [17]. SMS text messaging is also inexpensive, private, and can be scaled to large populations [16,17]. Thus, SMS text messaging could be an ideal approach for conducting mass depression screening for underserved, predominantly minority patients in safety net primary care systems.

Previous studies have tested the validity of conducting standardized depression screening, such as the Patient Health Questionnaire (PHQ), by using paper-based self-reported assessment [18-20], in-person interviewer (INTW) assessment [21,22], and telephone INTW assessment [11,21]. Patients with depression are at a higher risk of comorbid anxiety and functional disability; as many as 50% of depressed patients in the primary care setting suffer from anxiety and/or functional disability [8,23]. INTW-administered anxiety screening using the 2-item Generalized Anxiety Disorder (GAD-2) scale has been validated in 6 studies (reviewed by Plummer et al [24]). The INTW-administered functional disability assessment using the Sheehan Disability Scale (SDS) has been validated by Leon et al [25]. Few studies have examined the validity of technology-mediated assessment for depression and its related conditions such as anxiety and functional disability. Depression screening requires participants to self-disclose sensitive health information such as a sad mood, anhedonia, and eating and sleeping problems. Current evidence on the effect of technology being used to disclose such information is weak and inconsistent. It has been suggested that technology-mediated assessments, such as SMS text messaging, may help to create an idealized perception of the information collector and thus reduce social desirability bias [26]. This effect may encourage disclosure of sensitive health information [27,28]. In contrast, there is also evidence suggesting that technology-mediated assessments discourage disclosure of sensitive information as the distance and private space created by technology may discourage patients to seek help [29].

To fill in this knowledge gap, this study examined the validity of using standardized tools to assess depression and its related conditions via SMS text messaging vs the gold standard INTW assessment in underserved, predominantly minority patients from a large safety net primary care system. This study examined the internal consistency, test-retest reliability, and concordance of the 2 modes of assessment. Patient characteristics, including demographics such as age, gender, race/ethnicity, and marital status; technology use; and psychological traits such as personality, cognitive vulnerability of depression, and depression stigma were further examined in a regression analysis to explore their correlations with the differences in the 2 modes of assessment.

## Methods

### Study Design

This study protocol was approved by the Institutional Review Board of the University of Southern California and has been published in *JMIR Research Protocol* [30]. Underserved, predominantly minority safety net primary care patients were recruited and randomly assigned using a simple randomization method to 1 of the 4 study groups: SMS text messaging/INTW, INTW/SMS text messaging, SMS text messaging/SMS text messaging, or INTW/INTW. Participants in the SMS text messaging/INTW group received the SMS text messaging assessment in English or Spanish as chosen by the participant for depression and its related comorbid conditions, including anxiety and functional disability. Within 7 to 10 days following the SMS text messaging assessment, a bilingual INTW contacted the participant over telephone to repeat the same assessment. Participants in the INTW/SMS text messaging group first answered the INTW assessment over telephone; then, they replied to the SMS text messaging assessment within 7 to 10 days following the INTW assessment. Participants in the SMS text messaging/SMS text messaging and INTW/INTW groups received 2 SMS text messages and 2 INTW assessments each, respectively. The interval between the 2 assessments was 7 to 10 days. The choice of the interval between the 2 assessments was based on a widely cited study that examined the validity of INTW assessments conducted by telephone vs in-person assessments of depression [21]. A shorter interval could increase the likelihood of repeating the answer from the first assessment in the subsequent assessment, whereas a longer interval could increase the probability of change in the actual severity of depression.

The SMS text messaging/INTW and INTW/SMS text messaging groups were used to examine the concordance between the SMS text messaging and INTW assessments. The SMS text messaging/SMS text messaging and INTW/INTW groups were used to evaluate test-retest reliability. Validity of the INTW assessment has been established in prior studies [21,31]; thus, the INTW assessment served as the gold standard in this study. The participants for this study were recruited from the participants of the prior Diabetes-Depression Care-management Adoption Trial (DCAT), a large, US Department of Health and Human Services–funded translational study, in partnership with the Los Angeles County Department of Health Services, the second largest safety net system in the United States [11,32-39]. These patients were chosen from the DCAT due to prior contact and rapport built in the DCAT, and the study fit the timeline for the funding requirement. The inclusion criteria were as follows: (1) patients were DCAT participants, (2) possessed an SMS text messaging–capable phone, (3) knew how to send and receive SMS text messages, and (4) could speak and read English or Spanish. Patients unable to provide consent were excluded from the study.

As described in the study protocol paper [30], there is no consensus in the method to determine the sample size *a priori* for a validity study. Well-received published studies that evaluated the PHQ and the SDS in primary care using an INTW assessment typically had a sample size that ranged from 100 to more than 3000 [21,33,40,41]. Using the method developed by Walter et al [42], the sample size needed to evaluate the concordance using an intraclass correlation coefficient (ICC) was 80 to attain a type I error of .05 and a type II error of .20 based on the assumptions that the minimum acceptable concordance was 0.6 (ie, threshold of good concordance as suggested by Cicchetti [43]) and the expected concordance was 0.75. The sample size needed to evaluate test-retest reliability using ICC was 40 to attain a type I error of 0.05 and a type II error of 0.20 based on the assumptions that the minimum acceptable test-retest reliability was 0.6 and the expected reliability was 0.8. The targeted sample size of this study was set to 200 (ie, 50 participants in each of the 4 groups). This led to a total of 100 subjects (ie, 50 in the SMS text messaging/INTW group and 50 in the INTW/SMS text messaging group) to evaluate concordance and 50 subjects per mode of assessment to evaluate test-retest reliability.

### Measurements

The depression screening was conducted using the 2-item and 8-item PHQ (PHQ-2 and PHQ-8, respectively), which are widely used depression screening tools in primary care and general populations [27]. The PHQ-8 has 8 questions; each question uses a score of 0 to 3 to assess the frequency of a depressive symptom in the past 2 weeks. The total PHQ-8 score ranges from 0 to 24, with a higher score indicating severe depressive symptoms. A cutoff score of 8 has been suggested to identify major depression using the PHQ-8 [27]. The PHQ-2 comprises the first 2 questions of the PHQ-8. The PHQ-2 score ranges from 0 to 6, with PHQ-2≥3 indicating major depression [19]. Anxiety was assessed by the GAD-2 [44]. Each GAD-2 question uses a score of 0 to 3 to assess the frequency of an anxiety symptom in the past 2 weeks. The total GAD-2 score ranges from 0 to 6, with a higher score indicating severe anxiety symptoms. Functional disability was assessed by the SDS, which includes 3 questions to assess the degree of disruption (scored from 0 to 10) caused by health problems to work/school work, social life, and family life/home responsibilities [25]. The total SDS score ranges from 0 to 30, with a higher score indicating severe functional disability.

Participant characteristics included demographics (such as age, gender, race/ethnicity, language, marital status, and education), personality, cognitive diathesis to depression, depression stigma, and mobile phone use. Personality was measured by using the Ten-Item Personality measure of the Big Five personality scale: extraversion, agreeableness, conscientiousness, emotional stability, and openness to experience [45]. Cognitive diathesis to depression was measured by using the 9-item Dysfunctional Attitudes Scale (DAS)–Short Form [46]. The DAS measures 2 depression diatheses, ie, perfectionism and dependency, and has a score ranging from 0 to 3, with a higher score indicating higher depression diathesis. Depression stigma was measured by the Depression Stigma Scale (DSS) [47], which assesses both personal and perceived depression stigma. Both the personal and perceived DSS have a score range of 0 to 4, with a higher score indicating a higher stigma. Mobile phone usage was measured by recall questions for using the phone at least once per day during the past 2 weeks for the following functions: making a telephone call, sending or reading an SMS text message, using the internet, and using a mobile app. The number of mobile functions used daily by the participants was counted to generate a dichotomous variable indicating the use of three or more mobile functions. Using a mobile phone for health care was measured by recall questions asking if the mobile device was ever used for the following health care purposes: contacting a doctor, getting health information, and assistance with self-care. A dichotomous variable was generated to indicate whether the participant ever used a mobile phone for multiple health care purposes.

### Statistical Analysis

The participant characteristics were summarized using mean and standard deviation for continuous variables and frequency and percentage for dichotomous variables. The internal consistency was evaluated by using Cronbach alpha. The test-retest reliability of the SMS text messaging and INTW assessments was evaluated by using ICC. The concordance between the SMS text messaging and INTW assessments was evaluated by using ICC, a kappa statistic, an area under the receiver operating characteristic curve (AUROC), sensitivity, and specificity. ICC was used to measure the consistency or reproducibility of the SMS text messaging and INTW assessments. AUROC, sensitivity, and specificity were used to measure discriminative validity. The kappa statistic was used to measure interrater agreement. The kappa statistic, sensitivity, and specificity were computed using the threshold levels of PHQ-2 ≥3, PHQ-8 ≥8, GAD-2 ≥3, and SDS ≥12. The differences in the scores between the SMS text messaging and INTW assessments were summarized by using means and standard deviations. The differences were detected using a paired 2-tailed *t* test.

A regression analysis was conducted to further examine the associations between the participant characteristics and the differences in the scores between the SMS text messaging and INTW assessments. To identify the most predictive variables, all patient characteristics, as summarized in Table 1, were entered into a least absolute shrinkage and selection operator (LASSO) variable selection procedure [39,48]. LASSO is a regression-based variable selection method that introduces a penalization parameter, lambda, to a standard regression to penalize the size of the coefficient estimate. As the lambda value increases, the coefficient estimate shrinks toward 0 but at varying speeds. The shrinkage speed provides a way to rank the predictive power of each variable, as variables with a slower shrinkage speed are ranked with stronger predictive power. The top 4 predictive variables selected by LASSO were included in the linear regression models to estimate their associations with the differences in the scores between the SMS text messaging and INTW assessments. The goodness of fit of the linear regression models was evaluated using the original and adjusted measures.

All statistical analyses were conducted using R, version 3.5.2 (R Core team) [49]. Cronbach alpha and ICC were calculated using the *alpha* and *ICC* functions, respectively, in the R *psych* package [50]. The kappa statistic was evaluated using the *Kappa.test* function in the R *fmsb* package [51]. The AUROC was evaluated using the *roc* function in the R *pROC* package [52]. LASSO variable selection was conducted using the *glmnet* function in the R *glmnet* package [53]. Finally, the linear regression analysis was performed using the R *lm* function.

**Table 1:** Summary of the participant characteristics.

Variable		All (N=206)	SMS text messaging/INTW^a^ (n=52)	SMS text messaging/SMS text messaging (n=53)	INTW/SMS text messaging (n=49)	INTW/INTW (n=52)
Age (years), mean (SD)		57.11 (9.18)	58.54 (8.60)	55.35 (10.06)	57.24 (8.08)	57.33 (9.76)
Female, n (%)		119 (57.8)	33 (63.5)	34 (64.2)	26 (53.1)	26 (50.0)
Latino, n (%)		192 (93.2)	50 (96.2)	51 (96.2)	44 (91.7)	47 (92.2)
Preferred Spanish language, n (%)		160 (77.7)	39 (75.0)	47 (88.7)	38 (77.6)	36 (69.2)
Less than high-school level education, n (%)		131 (63.6)	31 (59.6)	33 (62.3)	35 (71.4)	32 (61.5)
Extraversion score, mean (SD)		3.84 (1.15)	3.84 (1.23)	4.03 (1.00)	3.68 (1.05)	3.81 (1.28)
Agreeableness score, mean (SD)		6.43 (0.87)	6.45 (0.84)	6.75 (0.53)	6.17 (0.95)	6.31 (1.01)
Conscientiousness score, mean (SD)		5.51 (1.48)	5.60 (1.24)	5.52 (1.73)	5.36 (1.56)	5.55 (1.37)
Emotional stability score, mean (SD)		5.47 (1.44)	5.37 (1.47)	5.47 (1.48)	5.43 (1.42)	5.61 (1.43)
Openness to experience score, mean (SD)		3.77 (1.23)	3.61 (1.38)	3.90 (1.15)	3.72 (1.24)	3.83 (1.17)
Dysfunctional attitude scale, mean (SD)		0.58 (0.70)	0.55 (0.65)	0.65 (0.76)	0.50 (0.66)	0.61 (0.74)
Personal depression stigma, mean (SD)		2.16 (1.08)	2.00 (1.12)	2.31 (1.11)	2.13 (0.96)	2.21 (1.13)
Perceived depression stigma, mean (SD)		3.20 (0.91)	3.07 (1.02)	3.40 (0.73)	3.14 (0.99)	3.16 (0.86)
**Mobile phone functions used every day (≥3), n (%)**		131 (63.6)	33 (63.5)	39 (73.6)	33 (67.3)	26 (50.0)
	0	3 (1.5)	0 (0.0)	0 (0.0)	1 (2.1)	2 (3.8)
	1	13 (6.4)	3 (5.8)	1 (1.9)	1 (2.1)	8 (15.4)
	2	56 (27.6)	16 (30.8)	12 (23.1)	12 (25.5)	16 (30.8)
	3	5 (2.5)	0 (0.0)	4 (7.7)	0 (0.0)	1 (1.9)
	4	126 (62.1)	33 (63.5)	35 (67.3)	33 (70.2)	25 (48.1)
**Mobile phone used for multiple health care purposes, n (%)**		86 (41.7)	22 (42.3)	26 (49.1)	22 (44.9)	16 (30.8)
	0 purposes	22 (10.8)	6 (11.5)	2 (3.8)	4 (8.3)	10 (19.2)
	1 purpose	96 (47.1)	24 (46.2)	24 (46.2)	22 (45.8)	26 (50.0)
	2 purposes	54 (26.5)	14 (26.9)	17 (32.7)	14 (29.2)	9 (17.3)
	3 purposes	32 (15.7)	8 (15.4)	9 (17.3)	8 (16.7)	7 (13.5)

^a^INTW: interviewer.

## Results

### Participant Characteristics

Participants were recruited from June 2017 to November 2017, which led to the enrollment of 206 participants: 52 in the SMS text messaging/INTW, 53 in the SMS text messaging/SMS text messaging, 49 in the INTW/SMS text messaging, and 52 in the INTW/INTW groups. The average age of the participants was 57.1 years, 57.8% (119/206) were females, and 93.2% (192/206) were Latinos. In addition, 77.7% (160/206) chose Spanish as their preferred language. Compared with the personality norms from a large sample [54], participants in this study were more agreeable (mean: this study=6.43; norm for males aged 51-60 years=4.89; and norm for females aged 51-60 years=5.43), more emotionally stable (mean: this study=5.47; norm for males aged 51-60 years=4.80; and norm for females aged 51-60 years=4.66), less open to new experiences (mean: this study=3.77; norm for males aged 51-60 years=5.39; and norm for females aged 51-60 years=5.42), similar in extraversion (mean: this study=3.84; norm for males aged 51-60 years=3.87; and norm for females aged 51-60 years=4.18), and similar in conscientiousness (this study=5.51; norm for males aged 51-60 years=5.11; and norm for females aged 51-60 years=5.35). Overall, 63.6% (131/206) of the participants used three or more mobile phone functions every day; only 41.7% (86/206) of the participants ever used a mobile phone for multiple health care purposes. Table 1 summarizes the participant characteristics.

### Internal Consistency and Test-Retest Reliability

The internal consistency and test-retest reliability of the INTW and SMS text messaging assessments were evaluated by using Cronbach alpha and ICC, respectively. As shown in Table 2, all measurements except the SMS text messaging–assessed PHQ-2 had Cronbach alpha values ≥.70. Following the guidelines [55,56], a Cronbach alpha value ≥.70 indicates greater than acceptable internal consistency. Both the INTW and SMS text messaging assessments for the PHQ-8 and SDS had Cronbach alpha values ≥.80, indicating good internal consistency [55,56]. All measurements except the INTW-assessed SDS had ICC values ≥0.75. Following the guidelines given by Cicchetti [43], these values indicate good to excellent test-retest reliability. The INTW-assessed SDS had an ICC value of 0.47, indicating fair test-retest reliability [43].

**Table 2:** Internal consistency and test-retest reliability of the interviewer and SMS text messaging assessments.

Assessment mode				Internal consistency (Cronbach alpha)		Test-retest reliability (intraclass correlation coefficient)
**Interviewer assessment**						
	**Depression**					
		PHQ-2^a^	.71		0.76	
		PHQ-8^b^	.86		0.78	
	Anxiety (GAD-2^c^)		.82		0.75	
	Functional disability (SDS^d^)		.80		0.47	
**SMS text messaging assessment**						
	**Depression**					
		PHQ-2	.68		0.74	
		PHQ-8	.86		0.81	
	Anxiety (GAD-2)		.71		0.73	
	Functional disability (SDS)		.86		0.82	

^a^PHQ-2: 2-item Patient Health Questionnaire.

^b^PHQ-8: 8-item Patient Health Questionnaire.

^c^GAD-2: 2-item Generalized Anxiety Disorder scale.

^d^SDS: Sheehan Disability Scale.

### Concordance

Table 3 summarizes the results of evaluating the concordance between the INTW and SMS text messaging assessments. The results show that the INTW-assessed depression and anxiety scores were lower on average than their paired SMS text messaging–assessed scores, indicating that people reported fewer symptoms of depression and anxiety via the INTW assessment than the SMS text messaging assessment. The INTW-assessed SDS scores were higher on average than their paired SMS text messaging–assessed scores, indicating that people reported more functional disability in the INTW assessment than the SMS text messaging assessment. Although paired *t* tests showed no significant differences in the mean scores, ICC and kappa statistic evaluations revealed some scales with poor concordance. ICC of the PHQ-2 was 0.32, indicating poor concordance between the INTW and SMS text messaging assessments [43]. ICC values of both the GAD-2 and the SDS were 0.54, suggesting fair concordance [43]. The PHQ-8 assessments had an ICC value of 0.73, indicating good concordance [43]. The kappa statistic suggested that the categorical agreements between the INTW and SMS text messaging assessments were poor for PHQ-2 ≥3 (kappa=0.19) and SDS ≥12 (kappa=0.13), following Landis and Koch [57]. The kappa statistic for GAD-2 ≥3 was 0.35, indicating fair agreement [57]. The kappa statistic for PHQ-8 ≥8 was 0.43, indicating moderate agreement [57]. The AUROC values were 0.84, 0.93, 0.76, and 0.94 for the PHQ-2, PHQ-8, GAD-2, and SDS, respectively. The sensitivity for the 3 shorter scales, ie, PHQ-2, GAD-2, and SDS, was <0.60, whereas the sensitivity for the PHQ-8 was 0.60. The specificity for all 4 scales was >0.85.

**Table 3:** Concordance between the interviewer and SMS text messaging assessments.

Measurement		Interviewer assessment, mean (SD)	SMS text messaging assessment, mean (SD)		*P*^a^ value	Intraclass correlation coefficient	Kappa value^b^	Area under the receiver operating characteristic curve	Sensitivity^b^	Specificity^b^
**Depression**										
	Patient Health Questionnaire (2-item)	0.67 (1.27)	1.23 (1.79)		.13	0.32	0.19	0.84	0.34	0.89
	Patient Health Questionnaire (8-item)	3.29 (4.47)	3.89 (4.20)		.39	0.73	0.43	0.93	0.60	0.86
Anxiety (2-item Generalized Anxiety Disorder scale)		0.97 (1.49)	1.16 (1.63)		.64	0.54	0.35	0.76	0.50	0.89
Functional disability (Sheehan Disability Scale)		8.09 (6.40)	6.83 (8.03)		.16	0.54	0.13	0.94	0.59	1.00

^a^*P* value was calculated by using a paired *t* test.

^b^The kappa statistic, sensitivity, and specificity were evaluated using a cutoff point of 3 for the 2-item Patient Health Questionnaire and 2-item Generalized Anxiety Disorder scale, 8 for the 8-item Patient Health Questionnaire, and 12 for the Sheehan Disability Scale.

### Associations Between the Participant Characteristics and the Differences in the Interviewer and SMS Text Messaging Assessment Scores

A regression analysis was performed to further examine the associations between the participant characteristics and the differences in the INTW and SMS text messaging assessment scores. Table 4 summarizes the results. Compared with the participants who were more conscientious, the less-conscientious participants were significantly associated with reporting more symptoms of depression (as assessed by the PHQ-2 and PHQ-8) and anxiety (as assessed by the GAD-2) in the INTW assessment than the SMS text messaging assessment. Compared with the more emotionally stable participants, the less emotionally stable participants were significantly associated with reporting fewer symptoms of depression (as assessed by the PHQ-2) and anxiety (as assessed by the GAD-2) in the INTW assessment than the SMS text messaging assessment. Compared with the participants who were not extremely agreeable, the extremely agreeable participants were significantly associated with reporting more depression symptoms (as assessed by the PHQ-2 and PHQ-8) in the INTW assessment than the SMS text messaging assessment. Compared with the participants who were open to new experiences, those who were less open to new experiences were significantly associated with reporting more functional disability (as assessed by the SDS) in the INTW assessment than the SMS text messaging assessment. All personality-related differences were non-negligible as the differences were >1 point for the PHQ-2 and GAD-2 (both have scores ranging from 0 to 6) and >2 points for the PHQ-8 (with a score ranging from 0 to 24). A personal depression stigma was significantly associated with reporting less depression (as assessed by the PHQ-8) and anxiety (as assessed by the GAD-2) in the INTW assessment than the SMS text messaging assessment. The only significant demographic variable was being married, which was significantly associated with reporting less depression (as assessed by the PHQ-8) in the INTW assessment than the SMS text messaging assessment. The *R*^2^ goodness of fit evaluation model showed that all regression models explained at least 40% of the variance in the data. The adjusted *R*^2^ values were >0.30 for all models. The regression model for the difference in PHQ-8 had the best goodness of fit, with *R*^2^=0.56 and adjusted *R*^2^=0.48. Diagnostic plots of the regression did not reveal any violation of the underlying assumptions of the model.

**Table 4:** Linear regression analysis using the top 4 predictors selected by least absolute shrinkage and selection operator to predict the differences between the interviewer and SMS text messaging assessments.

Predictors	Difference between interviewer and SMS text messaging assessments, estimate of coefficient (95% CI)			
	Patient Health Questionnaire (2-item)^a^	Patient Health Questionnaire (8-item)^b^	Generalized Anxiety Disorder scale (2-item)^c^	Sheehan Disability Scale^d^
Conscientiousness score ≤4.5	1.76 (0.58 to 2.94)^e^	2.39 (0.27 to 4.51)^e^	1.09 (0.09 to 2.05)^e^	−3.75 (−8.57 to 1.07)
Emotional stability score ≤4.5	−1.45 (−2.54 to −0.36)^e^	—^f^	−1.09 (−2.04 to −0.14)^e^	—
Agreeable score=7	1.33 (0.17 to 2.49)^e^	2.35 (0.38 to 0.32)^e^	—	2.74 (−1.88 to 7.36)
Openness to experience score ≥4.5	—	—	—	5.51 (0.50 to 10.51)^e^
Personal depression stigma	—	−0.94 (−1.87 to −0.10)^e^	−0.50 (−0.98 to −0.02)^e^	—
Dysfunctional attitude score	—	—	−0.36 (−1.14 to 0.42)	—
Married	—	−2.37 (−4.39 to −0.34)^e^	—	—
Gender	0.62 (−0.50 to 1.74)	—	—	1.76 (−2.75 to 6.26)

^a^*R*^2^ value=0.46, adjusted; *R*^2^ value=0.38

^b^*R*^2^ value=0.56, adjusted; *R*^2^ value=0.48

^c^*R*^2^ value=0.44, adjusted; *R*^2^ value=0.36

^d^*R*^2^ value=0.40, adjusted; *R*^2^ value=0.31

^e^*P*<.05.

^f^Some cells are empty because the corresponding variables are not selected into the regression model.

## Discussion

### Principal Findings

This study examined the validity of screening depression and related comorbid conditions, including anxiety and functional disability via the SMS text messaging and INTW assessments for underserved, predominantly minority safety net primary care patients. Although the longer PHQ-8 depression screening scale had good internal consistency, test-retest reliability, and concordance, the 3 shorter scales, ie, the PHQ-2, GAD-2, and SDS, had poor-to-moderate levels of concordance between the SMS text messaging and INTW assessments. In particular, the PHQ-2 depression screening scale had poor concordance, as measured by ICC and Cohen kappa, between the SMS text messaging and INTW assessments. The kappa value of the SDS also indicated poor agreement. The interrater agreement as measured using Cohen kappa would improve if different cutoff points were assigned based on the modes of assessment. The kappa value for the PHQ-2 depression screening scale would improve from 0.19 (indicating poor agreement) to 0.52 (indicating moderate agreement) if the cutoff points were changed from 3 for both modes of assessment to 2 for the INTW assessment and to 3 for the SMS text messaging assessment. Similarly, the kappa value for the SDS would improve from 0.13 (indicating poor agreement) to 0.49 (indicating moderate agreement) if the cutoff points were changed from 12 for both modes of assessment to a cutoff point of 12 for the INTW assessment and 9 for the SMS text messaging assessment.

This study found that participants reported more symptoms of depression and anxiety via the SMS text messaging assessment than the INTW assessment. In contrast, less functional disability was reported via the SMS text messaging assessment than the INTW assessment. The regression analysis revealed that a higher level of personal depression stigma was associated with reporting more symptoms of depression and anxiety via the SMS text messaging assessment than the INTW assessment. This finding supports the hypothesis that SMS text messaging creates a private and secure environment with less social desirability bias and therefore encourages people to self-report stigmatized symptoms of depression and anxiety [26-28]. The analysis also identified that the differences in the scores between the SMS text messaging and INTW assessments were associated with personality traits. Personality traits refer to habitual patterns of behavior, thoughts, and emotions that are relatively stable over time, are relatively consistent among situations, and influence behavior [58]. As few studies have examined the relationship between personality traits and self-reporting of sensitive health information, it is difficult to judge whether these findings imply causal relationships or merely a statistical association. The finding that a higher level of conscientiousness was related to reporting more symptoms of depression and anxiety via the SMS text messaging assessment than the INTW assessment may be explained by the nature of conscientious participants, who tended to be careful, diligent, and deliberate and who were better at retrieving and reporting symptoms of depression and anxiety in the more private, less time-pressured and less socially biased environment created by the SMS text messaging assessment. A lower level of emotional stability appeared to be associated with reporting more symptoms of depression and anxiety via the SMS text messaging assessment than the INTW assessment. This may be explained by the fact that people with a lower level of emotional stability tend to have a higher level of negative emotional experience [59] such as sadness and fear, which are core symptoms of depression and anxiety measured by the PHQ-2 and GAD-2. The less-pressured environment of the SMS text messaging assessment may facilitate better retrieval and reporting of these negative emotional experiences. To the best of our knowledge, no study has reported a reasonable explanation for the correlations between agreeableness and openness to new experience and the differences in reporting via the SMS text messaging and INTW assessments. Those correlations may be caused by some unknown mechanism or merely randomness in the data. Finally, the regression analysis identified that unmarried participants reported more depressive symptoms via the INTW assessment than the SMS text messaging assessment. This phenomenon may be explained by unmarried participants who may be more likely to use the INTW assessment to seek help by reporting more symptoms [29], whereas married participants were less likely to do so as they may have stronger social network support. Overall, the regression analysis suggested that people may self-report sensitive health information differently to technology-mediated assessment than INTW assessment modes based on their personality traits, stigma with depression, and certain demographic characteristics.

### Limitations

This study had a few limitations that should be discussed. First, the study participants’ experience built in the prior DCAT study may have made those participants more familiar with technology-mediated assessments than the average person in the targeted study population. Nevertheless, the 4-year interval between the DCAT study (conducted during 2010-2013) and this study (conducted in 2017) was not short and thus is likely to decrease the potential influence of the DCAT assessment. Second, the study participants were predominantly Latinos, which may limit the generalizability of the results to other safety net primary care populations, particularly those of African American patients. Finally, the statistical associations revealed by the regression analysis need further exploration for the causal mechanism underlying self-reporting sensitive health information via different modes of assessment.

### Conclusions

This study examined the validity of screening depression and related conditions via an SMS text messaging vs interview assessment for underserved, predominantly minority safety net primary care patients. The depression screening conducted using the longer PHQ-8 scale via SMS text messaging demonstrated good internal consistency, test-retest reliability, and concordance with the gold standard INTW assessment mode. Deploying shorter scales via SMS text messaging should be done cautiously. A further regression analysis supported that technology-mediated assessments, such as SMS text messaging, may create a private space with less pressure from personal depression stigma and therefore encourage self-disclosure of depressive symptoms. Other characteristics such as personality traits and certain demographic characteristics were also associated with the difference between technology-mediated and INTW assessment modes.

## Abbreviations

AUROC: area under the receiver operating characteristic curve
DAS: Dysfunctional Attitudes Scale
DCAT: Diabetes-Depression Care-management Adoption Trial
DSS: Depression Stigma Scale
GAD: Generalized Anxiety Disorder
ICC: intraclass correlation coefficient
INTW: interviewer
LASSO: least absolute shrinkage and selection operator
PHQ: Patient Health Questionnaire
SDS: Sheehan Disability Scale
